# Distinct genetic control of autoimmune neuropathy and diabetes in the non-obese diabetic background

**DOI:** 10.1016/j.jaut.2013.06.005

**Published:** 2013-07-12

**Authors:** Hélène Bour-Jordan, Heather L. Thompson, Jennifer R. Giampaolo, Dan Davini, Wendy Rosenthal, Jeffrey A. Bluestone

**Affiliations:** University of California in San Francisco, 513 Parnassus Avenue, Box 0400, San Francisco, CA 94143-0400, USA

**Keywords:** NOD-B7-2KO mice, Autoimmunity, Peripheral neuropathy, Diabetes, *Idd* loci, Tregs

## Abstract

The non-obese diabetic (NOD) mouse is susceptible to the development of autoimmune diabetes but also multiple other autoimmune diseases. Over twenty susceptibility loci linked to diabetes have been identified in NOD mice and progress has been made in the definition of candidate genes at many of these loci (termed *Idd* for insulin-dependent diabetes). The susceptibility to multiple autoimmune diseases in the NOD background is a unique opportunity to examine susceptibility genes that confer a general propensity for autoimmunity versus susceptibility genes that control individual autoimmune diseases. We previously showed that NOD mice deficient for the costimulatory molecule B7-2 (NOD-B7-2KO mice) were protected from diabetes but spontaneously developed an autoimmune peripheral neuropathy. Here, we took advantage of multiple NOD mouse strains congenic for *Idd* loci to test the role of these *Idd* loci the development of neuropathy and determine if B6 alleles at *Idd* loci that are protective for diabetes will also be for neuropathy. Thus, we generated NOD-B7-2KO strains congenic at *Idd* loci and examined the development of neuritis and clinical neuropathy. We found that the NOD-H-2^g7^ MHC region is necessary for development of neuropathy in NOD-B7-2KO mice. In contrast, other *Idd* loci that significantly protect from diabetes did not affect neuropathy when considered individually. However, we found potent genetic interactions of some *Idd* loci that provided almost complete protection from neuritis and clinical neuropathy. In addition, defective immunoregulation by Tregs could supersede protection by some, but not other, *Idd* loci in a tissue-specific manner in a model where neuropathy and diabetes occurred concomitantly. Thus, our study helps identify *Idd* loci that control tissue-specific disease or confer general susceptibility to autoimmunity, and brings insight to the Treg-dependence of autoimmune processes influenced by given *Idd* region in the NOD background.

## 1. Introduction

The non-obese diabetic (NOD) mouse strain is a prototypic murine model of type 1 diabetes that has served as an important tool for dissecting mechanisms involved in the breakdown of immune tolerance in autoimmune diabetes [1]. In addition to diabetes, NOD mice are prone to development of other autoimmune syndromes including: autoimmune sialadenitis [2], autoimmune thyroiditis [3], autoimmune peripheral polyneuropathy [4], prostatitis in male mice [5] and some features of non-organ-specific autoimmune disease such as hemolytic anemia and late-onset anti-nuclear antibodies, a systemic lupus erythematosus (SLE)-like disease if exposed to killed mycobacterium [5–7]. Autoimmune diseases in NOD mice share many similarities to the comparable human diseases, including the presence of organ-specific autoantibodies, autoreactive CD4^+^ and CD8^+^ T cells, and genomic synteny of susceptibility loci. Thus, the NOD mouse has been considered as a good model for other autoimmune diseases such as Sjogren’s Syndrome, Guillain–Barre Syndrome and MS [4,8,9].

Autoimmune diabetes in the NOD mouse is polygenically controlled by as many as two dozen loci (termed *Idd* for insulin-dependent diabetes) distributed over 14 chromosomes [10–12]. NOD congenic strains have been developed that contain NOD genome at all loci except for one (or a few) “introgressed” allelic variants from a non-autoimmune mouse strain, usually C57BL/6 (B6) or C57BL/10 (B10) mice. These congenic models have been instrumental for the definition of genes and pathways that contribute to susceptibility to autoimmune diabetes. *Idd1* was one of the first loci to be identified in the NOD mouse and spans the major histocompatibility complex (MHC) region of the NOD mouse [13–17]. NOD mice harbor a unique MHC haplotype, termed H-2^g7^, which is necessary for the development of diabetes and represents the highest genetic contributor to disease susceptibility [18]. The exact gene or regulatory elements that are responsible for the effects of other *Idd* loci on disease still await identification but candidate genes have been identified for several *Idd* loci that are strong contributors to diabetes [10,11]. Besides the MHC, *Idd3* on chromosome 3 disproportionately contributes to the development of disease. NOD mice congenic for the *Idd3* locus have a strongly reduced incidence of diabetes and candidate genes include IL-2 and IL-21 [19–24]. Other individual *Idd* loci alter the development of diabetes to various degrees but interactions of protective alleles can confer stronger protection against diabetes in NOD mice. For example, introgressed B6 alleles for *Idd*5 or *Idd*10/*Idd*18 are almost fully protective for insulitis and diabetes when combined with the B6 *Idd3* region but they have more modest effects in isolation [23,25–28].

We previously showed that NOD mice deficient for the costimulatory molecule B7-2 (NOD-B7-2KO mice) were protected from autoimmune diabetes and sialadenitis but spontaneously developed autoimmune peripheral polyneuropathy [4]. NOD-B7-2KO mice exhibit limb paralysis associated with severe demyelination in the peripheral nerves beginning at 20–25 wk of age, and the disease affected 80–100% of NOD-B7-2KO females and 30–40% males by 30–35 wk of age [4]. Peripheral neuropathy in NOD-B7-2KO mice is dependent on interferon-gamma (IFN-γ)-producing CD4^+^ T cells that include autoreactive CD4^+^ T cells specific for peripheral nerve antigens such as myelin protein zero (P0) [4,29,30]. Autoimmune peripheral polyneuropathy has also been described in NOD mice after disruption of various pathways involved in immune tolerance such as IL-2, PD-1 or the autoimmune regulator (Aire) and appears to have comparable immunopathogenic properties as the NOD-B7-2KO disease [31–33]. In particular, NOD mice partially deficient in Aire function develop peripheral neuropathy that is mediated by CD4+ T cells targeting myelin P0 and IFN-γ is required for disease to develop [33–35], similar to what has been observed in NOD-B7-2KO mice [4,29,30]. In contrast, peripheral poly-neuropathy does not occur in mice deficient for B7-2, Aire or PD-1 on B6 or mixed B6-129 backgrounds. This suggested that development of autoimmune peripheral neuropathy on the NOD background may be related to its genetic propensity to autoimmunity. Additionally, T cell responses and autoAbs directed at Schwann cells surrounding the pancreatic islets of Langerhans have been detected in NOD mice, and it was suggested that the insulitis that precedes overt clinical diabetes may be, in part, directed against this peri-islet Schwann cell network. NOD mice have also been shown to be susceptible to autoimmunity targeting the central nervous system and can develop experimental autoimmune encephalomyelitis (EAE) [9,36]. Taken together, these data raised the possibility that common genes and pathways may be implicated in autoimmune pathologies targeting the pancreatic islets and the nervous system in the NOD mouse. In this study, we examined this question by focusing on loci previously shown to confer susceptibility to autoimmune diabetes in NOD mice by crossing NOD-B7-2KO to selected NOD congenic strains in order to compare the genetic control of autoimmune diabetes versus neuropathy. The studies showed that there is only a partial overlap in the genetic control of diabetes versus neuropathy in the NOD background.

## 2. Materials and methods

### 2.1. Mice

NOD-B7-2KO mice have been described previously [4]. NOD mice congenic for *Idd1* (H-2^h4^ and H-2^b^), *Idd3*, *Idd5*, *Idd9*, and *Idd10*/*18* were obtained from Taconic. NOD mice congenic for *Idd4* [37] were generously provided by Qing-Sheng Mi (Henry Ford Immunology Program, Detroit, MI). NOD-B7-2KO mice were crossed to individual NOD-*Idd* congenic mice and F1 mice (heterozygous for B7-2 and *Idd* alleles) were intercrossed to generate NOD-B7-2KO-*Idd* congenic mice. The genotype for B7-2 was determined by PCR of tail DNA as described [4]. The genotype for *Idd1* was determined by flow cytometry of PBMCs isolated from the tail vein using mAbs against H-2K^d^ and I-A^g7^ (NOD allele), H-2K^k^ (H-2^h4^ allele) and H-2K^b^ (H-2^b^ allele). The genotype for other *Idd* loci was determined by PCR using primer pairs specific for DNA segments including *Idd3* (ATGAGTTGGGAAGCTTGTGC and GTAAAGGCCAAGGGAAAAGG for marker D3nds36), *Idd4* (TAA-GAACCTTCTGTAGTTATT and ACCTTAGTTAGAGTTGGTCTC for marker D11Nds1; TTTCATGACCCCTAATTTCCC and GTGGGTGTGCCTGTCAATC for marker D11Mit39), *Idd5* (TCTAGGTTCCTGGGATAGAATCC and ATAGAAGCAGACCCAGAAGCC for marker D1Mit74; TATTGTTTATG-GAAATTGGACCC and CATCTCTGAAGGAAAAAGTGCA for marker D1Mit132), *Idd9* (TGGTCATGTGTGTCCATGC and ACTTCATGTAGC-CAGGTGGG for D4Mit233; GACAAACCACATGTAATGTGTGG and CTGCCTGCAGGCTGTATGTA for marker D4Mit180), and *Idd10*/*18* (TAGACCAATCTTGGGAGTGTCC and GGAAAAGCATAAGAAACAACCG for marker D3Mit12; ATCTGAGCAATCCAGAGTTAGTCA and GCAACCT CTGCATGCATG for marker D3Mit300). Indicated mice were treated with 50 μg anti-B7-1 mAbs every other day for 14 days between 2 and 4 weeks of age. Neuropathy and diabetes were assessed weekly as previously described [4,29]. Only female mice were used except where indicated. All mice were housed in a specific pathogen-free facility at The University of California at San Francisco. All animal experiments were approved by the Institutional Animal Care and Use Committee of the University of California, San Francisco.

### 2.2. Histology

For histological analysis, tissues were fixed in formalin and embedded in paraffin. Multiple 5 μm sections were stained with hematoxylin and eosin. For scoring of sciatic nerve and thyroid gland infiltration, scores of 0, 1, 2 and 3 indicate no, mild, moderate and severe lymphocytic infiltration, respectively.

### 2.3. In vitro cytokine production

Single-cell suspensions were prepared from spleen and LN of indicated mice. DMEM-glutamax medium (Life Technologies, Gaithersburg, MD) supplemented with 5% heat-inactivated FCS (Summit Biotechnology, Ft. Collins, CO), 100 U/ml penicillin, 100 U/ml streptomycin, nonessential amino acids, 10 mM HEPES and 50 μM β-mercaptoethanol (all from Life Technologies) was used for cell culture. For primary stimulation, spleen and LN cells (2 × 10^5^) were stimulated with anti-CD3 (0.1 or 1 μg/ml) alone or together with anti-CD28 (1 μg/ml) mAbs. For secondary stimulation, cells were stimulated with anti-CD3 (1 μg/ml) and anti-CD28 (1 μg/ml) mAbs for 7 days, with addition of 20 U/ml recombinant human IL-2 on day 3. On day 7, cells were restimulated with anti-CD3 with or without anti-CD28 mAbs as above. Supernatant was harvested from triplicate cultures after one (IL-2) or two (IFN-γ) days for both primary and secondary stimulation. Levels of cytokine were measured by commercial ELISA kits according to the manufacturer’s recommendations (BD-PharMingen, San Diego, CA).

### 2.4. Statistical analysis

All statistical analyses were performed using GraphPad Prism version 5.04. Statistical comparison of cumulative incidence curves was performed using a Log-Rank (Mantel–Cox) test. Statistical comparison of infiltration severity was performed using an unpaired two-tailed Mann–Whitney test. For all tests, *p* values < 0.05 were considered significant.

## 3. Results

### 3.1. The NOD-H-2^g7^ MHC region is necessary for the development of autoimmune peripheral neuropathy

**Fig. 1:**
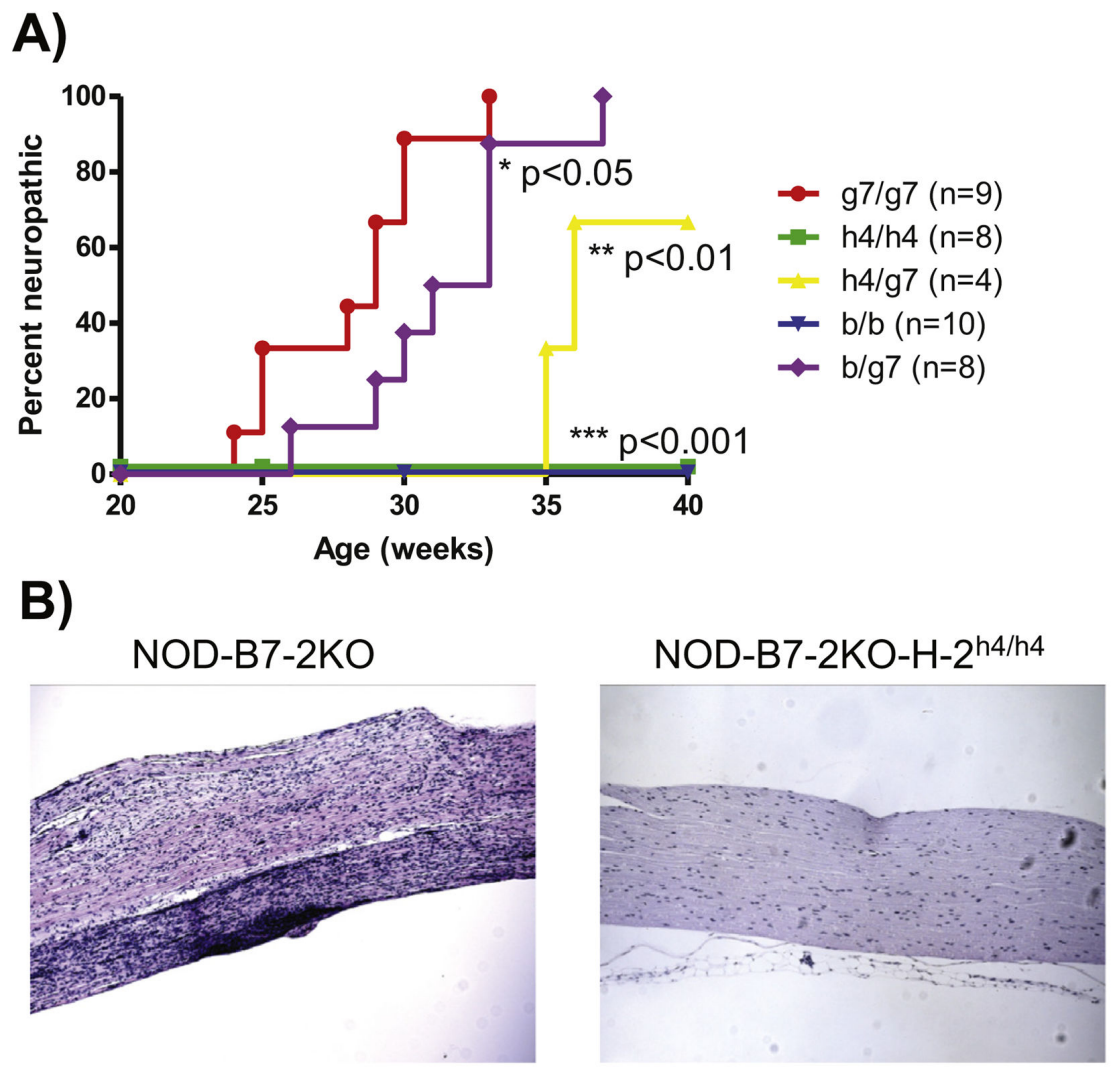
The NOD H-2^g7^ MHC locus is necessary for autoimmune peripheral neuropathy in NOD-B7-2KO mice. NOD-B7-2KO mice (H-2^g7/g7^) were crossed to NOD-H-2^h4/h4^ or NOD-H-2^b/b^ congenic mice to generate NOD-B7-2KO-H-2^h4/h4^ and NOD-B7-2KO-H-2^b/b^ mice. A) Congenic NOD-B7-2KO mice that were homozygous or heterozygous for H-2^g7^, H-2^h4^ or H-2^b^ were followed for the development of neuropathy. Cumulative incidence of neuropathy is shown for females of indicated genotypes. Incidence curves significantly different from incidence in NOD-B7-2KO mice are indicated (Log-rank (Mantel–Cox) test). B) H&E staining of sciatic nerves isolated from NOD-B7-2KO and NOD-B7-2KO-H2^h4/h4^ mice.

The H-2^g7^ MHC region is the only locus that is required for the development of autoimmune diabetes in NOD mice. NOD-H-2^h4^ congenic mice that express H-2K^k^ and I-A^k^ MHC class I and class II alleles are completely protected from diabetes [38]. We crossed NOD-B7-2KO mice to NOD-H-2^h4^ mice to generate NOD-B7-2KO-H-2^h4^ mice and followed these congenic mice for the development of neuropathy. We found that NOD-B7-2KO-H-2^h4/h4^ females were completely protected from autoimmune peripheral neuropathy (Fig. 1A) in comparison with NOD-B7-2KO-H-2^g7/g7^ littermates and NOD-B7-2KO mice which all developed neuropathy by 35 weeks of age (Fig. 1A and data not shown). Histological analysis of sciatic nerves showed an absence of mononuclear infiltration in peripheral nerves of NOD-B7-2KO-H-2^h4/h4^ mice, in contrast with the severe infiltration observed in NOD-B7-2KO mice (Fig. 1B). The absence of mononuclear infiltrate in NOD-B7-2KO-H-2^h4/h4^ mice is not the result of delayed kinetics of disease since sciatic nerves were still free of infiltration in >65 week-old NOD-B7-2KO-H-2^h4/h4^ mice (data not shown). Surprisingly, autoimmune neuropathy could develop in NOD-B7-2KO-H-2^h4/g7^ heterozygous mice, albeit disease was significantly delayed in NOD-B7-2KO-H-2^h4/g7^ mice compared with NOD-B7-2KO^g7/g7^ controls (Fig. 1A). This is notably different from autoimmune diabetes since the MHC region must be homozygous for H-2^g7^ for diabetes to develop in NOD mice [13,16,17].

To determine if this result was specific to the H-2^h4^ haplotype or could be generalized to other MHC haplotypes, we crossed NOD-B7-2KO mice to NOD-H-2^b^ congenic mice and followed NOD-B7-2KO-H-2^b^ female mice for autoimmune neuropathy. In agreement with the results obtained after introgression of H-2^h4^ MHC alleles, NOD-B7-2KO mice homozygous for H-2^b^ MHC alleles were completely protected from peripheral neuropathy (Fig. 1A). Moreover, 100% of NOD-B7-2KO-H-2^b/g7^ heterozygous mice developed neuropathy although kinetics of disease were significantly delayed compared to NOD-B7-2KO mice (*p* < 0.05). The development of neuropathy was not significantly different in NOD-B7-2KO-H-2^b/g7^ vs. NOD-B7-2KO-H-2^h4/g7^ mice (*p* = 0.08). Taken together, these data demonstrate that MHC-linked diabetogenic genes of the NOD mouse are also required for the development of autoimmune peripheral neuropathy in NOD-B7-2KO mice. However, unlike the development of diabetes in this setting, the NOD H-2^g7^ MHC locus is dominant in the control of autoimmune neuropathy.

### 3.2. Individual Idd regions that contribute to susceptibility to diabetes in NOD mice do not control the development of neuropathy in NOD-B7-2KO mice

**Fig. 2:**
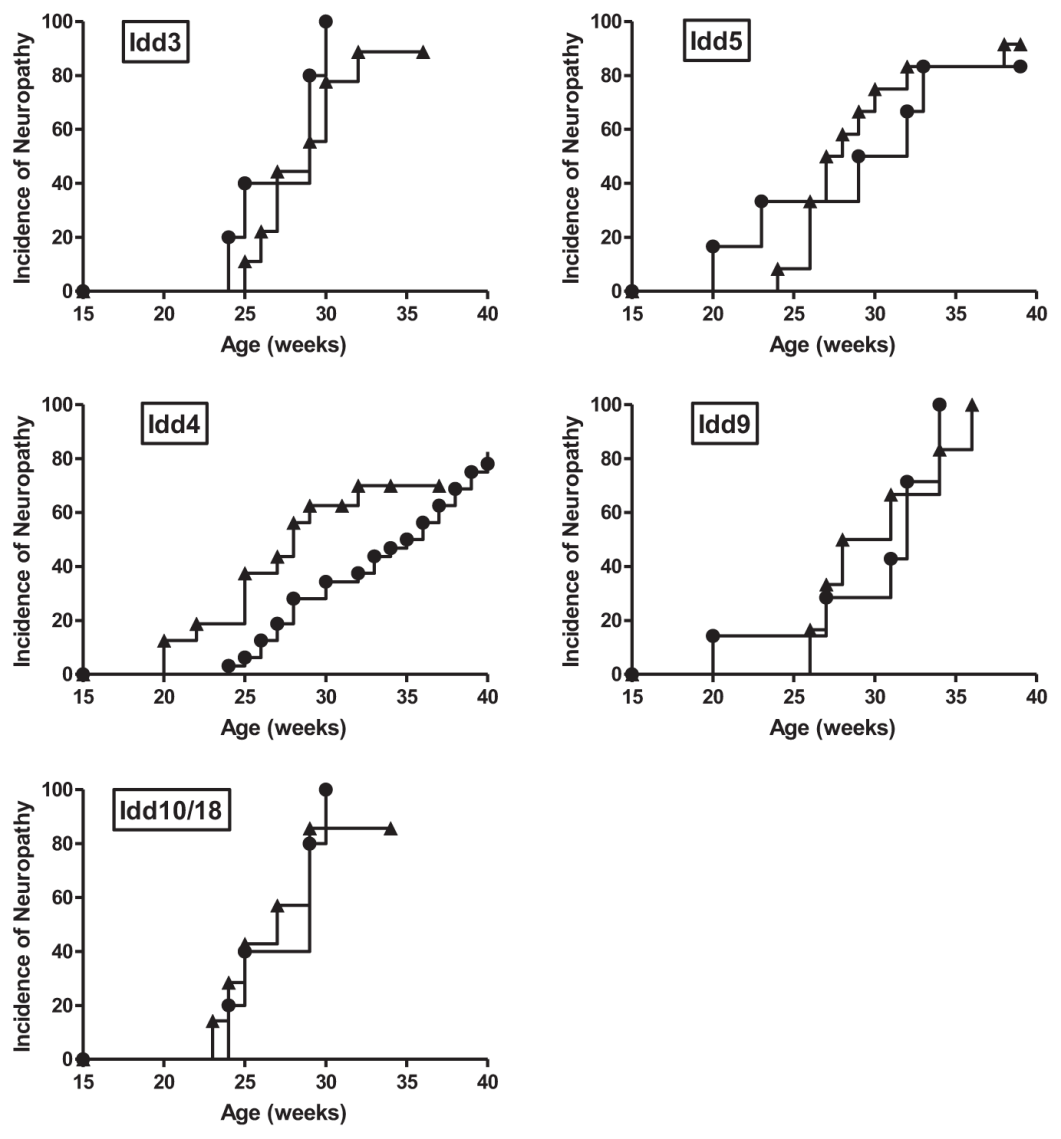
Diabetes-protective alleles at individual Idd loci do not alter the course of autoimmune peripheral neuropathy in NOD-B7-2KO mice. NOD-B7-2KO mice were crossed to NOD mice congenic for individual Idd loci to create congenic NOD-B7-2KO strains that carry introgressed alleles from diabetes-resistant B6, B10 or NOR mice at indicated Idd loci. Introgressed alleles at these loci were individually shown to reduce diabetes incidence by ~40–100% in NOD congenic strains [10–12]. Cumulative incidence of neuropathy is shown for NOD-B7-2KO females congenic for indicated Idd loci (triangles) and relevant cohorts of NOD-B7-2KO controls (circles).

We wished to determine if alleles at *Idd* loci other than the MHC region that are protective for diabetes in NOD mice would also reduce or delay the incidence of neuropathy in NOD-B7-2KO mice. For these studies, we selected *Idd* loci that were previously shown to individually afford some level of resistance to diabetes as demonstrated by significantly reduced and/or delayed diabetes incidence in NOD mice congenic for these intervals. Thus, NOD-B7-2KO mice were crossed to NOD mice congenic for *Idd3*, *Idd4*, *Idd5*, *Idd9* or *Idd10*/*18* regions and resulting congenic NOD-B7-2KO females were followed for the development of neuropathy. Of note, we used NOD congenic mice with wide intervals in *Idd* regions containing several loci in order to include all susceptibility genes in our analysis. Namely, *Idd4* encompasses *Idd4.1* and *Idd4.2* [37], *Idd5* includes *Idd5.1*, *Idd5.2* and *Idd5.3* [39,40], *Idd9* encompasses *Idd9.1*, *Idd9.2* and *Idd9.3* [41–43], and *Idd10*/*18* includes closely linked *Idd10* and *Idd18* on chromosome 3 [44,45]. Our data showed that there was no significant difference in the development of neuropathy in NOD-B7-2KO mice congenic for any of these loci as compared to NOD-B7-2KO mice (Fig. 2). Thus, whereas alleles at *Idd3*, *Idd4*, *Idd5*, *Idd9*, or *Idd10*/*18* loci introgressed from diabetes-resistant B6, B10 or NOR strains reduced the incidence of diabetes in NOD mice, they did not confer any protection to NOD-B7-2KO mice for development of autoimmune neuropathy.

### 3.3. Idd3/5 and Idd3/10/18 combinations partially protect NOD-B7-2KO mice from neuropathy

**Fig. 3:**
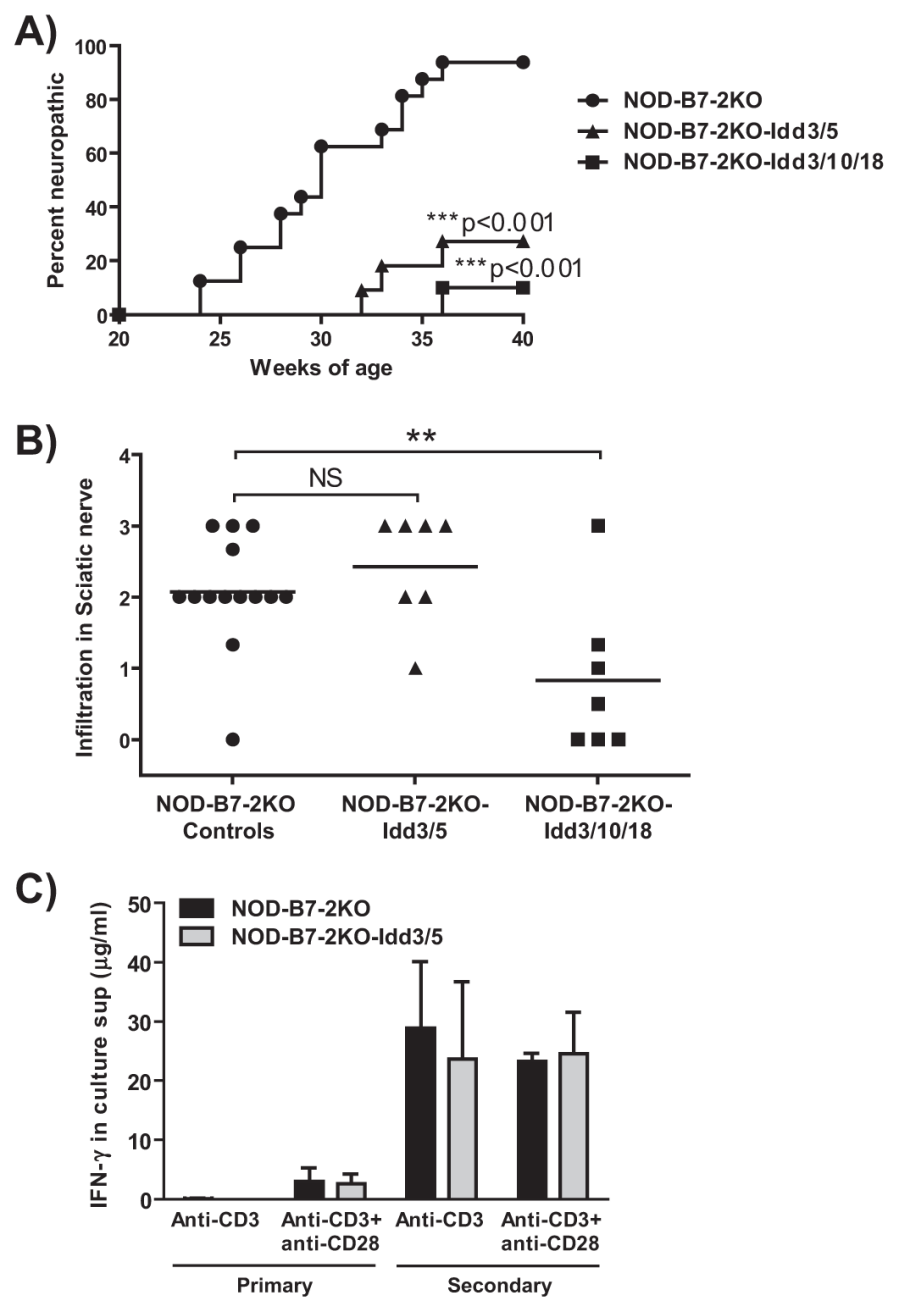
Differential effect of *Idd3*/*5* and *Idd3*/*10*/*18* combinations on clinical disease and tissue infiltration in NOD-B7-2KO mice. NOD-B7-2KO mice congenic for *Idd3* were crossed to NOD-B7-2KO mice congenic for *Idd5* or *Idd10*/*18* to generate NOD-B7-2KO mice congenic for both *Idd3* and *Idd5* (NOD-B7-2KO-*Idd3*/*5*) or *Idd3* and *Idd10*/*18* (NOD-B7-2KO-*Idd3*/*10*/*18*). A) Congenic females and NOD-B7-2KO control females were followed for neuropathy as in Fig. 1. B) Sciatic nerves were isolated from age-matched mice in indicated strains, stained with H&E and scored for infiltration as indicated in Materials and methods. Statistical comparison of infiltration severity was performed using the Mann–Whitney test (NS: non-significant; *: *p* = 0.017). C) Spleen and LN cells of NOD-B7-2KO-*Idd3*/*5* congenic mice and NOD-B7-2KOcontrols were stimulated with anti-CD3+/− anti-CD28 mAbs in primary or secondary stimulations in vitro and IFN-γ production was measured by ELISA of culture supernatants.

It was previously shown in NOD mice that protective alleles at given *Idd* loci could have an additive or synergistic effect when they were concurrently introgressed in the NOD genome. In particular, NOD mice congenic for *Idd3*/*Idd5* or *Idd3*/*Idd10*/*Idd18* were almost completely protected from insulitis and diabetes whereas mice congenic for *Idd3*, *Idd5*, *Idd10* or *Idd18* alone were not [23,25–28]. Thus, we intercrossed NOD-B7-2KO mice congenic for *Idd3* to NOD-B7-2KO mice congenic for *Idd5* or *Idd10*/*18*. NOD-B7-2KO mice congenic for *Idd3* and *Idd5* (NOD-B7-2KO.*Idd3*^b6/b6^.*Idd5*^b10/b10^) will hereafter be referred to as NOD-B7-2KO-*Idd3*/*5* mice, NOD-B7-2KO mice congenic for *Idd3* and *Idd10*/*18* (NOD-B7-2KO.*Idd3*^b6/b6^.*Idd10*/*18*^b6/b6^) as NOD-B7-2KO-*Idd3*/*10*/*18* mice, and NOD-B7-2KO controls (NOD-B7-2KO.*Idd*^nod/nod^) as NOD-B7-2KO mice. Our data showed that B6/B10 alleles at *Idd3* and *Idd5* or *Idd10*/*18* had a synergistic protective effect on autoimmune peripheral neuropathy since NOD-B7-2KO-*Idd3*/*5* and NOD-B7-2KO-*Idd3*/*10*/*18* females were almost completely protected from disease (Fig. 3A). Although the incidence of neuropathy was not statistically different between NOD-B7-2KO-*Idd3*/*5* and NOD-B7-2KO-*Idd3*/*10*/*18* mice, histological analyses revealed distinct levels of sciatic nerve infiltration in the two congenic strains (Fig. 3B). Indeed, infiltration in sciatic nerves of NOD-B7-2KO-*Idd3*/*5* was as severe as in NOD-B7-2KO mice whereas infiltration was significantly reduced in NOD-B7-2KO-*Idd3*/*10*/*18* mice.

Since IFN-γ is required for the development of neuropathy in NOD-B7-2KO mice [29], we determined if the reduced incidence of clinical disease despite a strong infiltrate in peripheral nerves of NOD-B7-2KO-*Idd3*/*5* mice could be due to defective IFN-γ production by T cells. Spleen and LN cells were isolated from age-matched NOD-B7-2KO and NOD-B7-2KO-*Idd3*/*5* mice and stimulated *in vitro* with anti-CD3 mAbs with or without anti-CD28 mAbs. Production of IFN-γ after primary or secondary stimulation was measured in culture supernatants by ELISA. We could not detect any difference in IFN-γ production by NOD-B7-2KO versus NOD-B7-2KO-*Idd3*/*5* T cells (Fig. 3C). IL-2 production was also comparable in NOD-B7-2KO and NOD-B7-2KO-*Idd3*/*5* mice (data not shown). Similar IFN-γ production by NOD-B7-2KO versus NOD-B7-2KO-*Idd3*/*5* T cells was observed in all conditions tested, *i.e.* after primary and secondary stimulation, after stimulation with anti-CD3 mAbs in the presence or absence of anti-CD28 mAbs, and using anti-CD3 mAbs at different concentrations (Fig. 3C and data not shown). Thus, protection from disease in NOD-B7-2KO-*Idd3*/*5* mice does not appear to result from a defect in IFN-γ production.

### 3.4. Tissue- and sex-specific effects of Idd3/5 and Idd3/10/18 combinations on disease are revealed in NOD-B7-2KO mice in conditions of defective immunoregulation

**Fig. 4:**
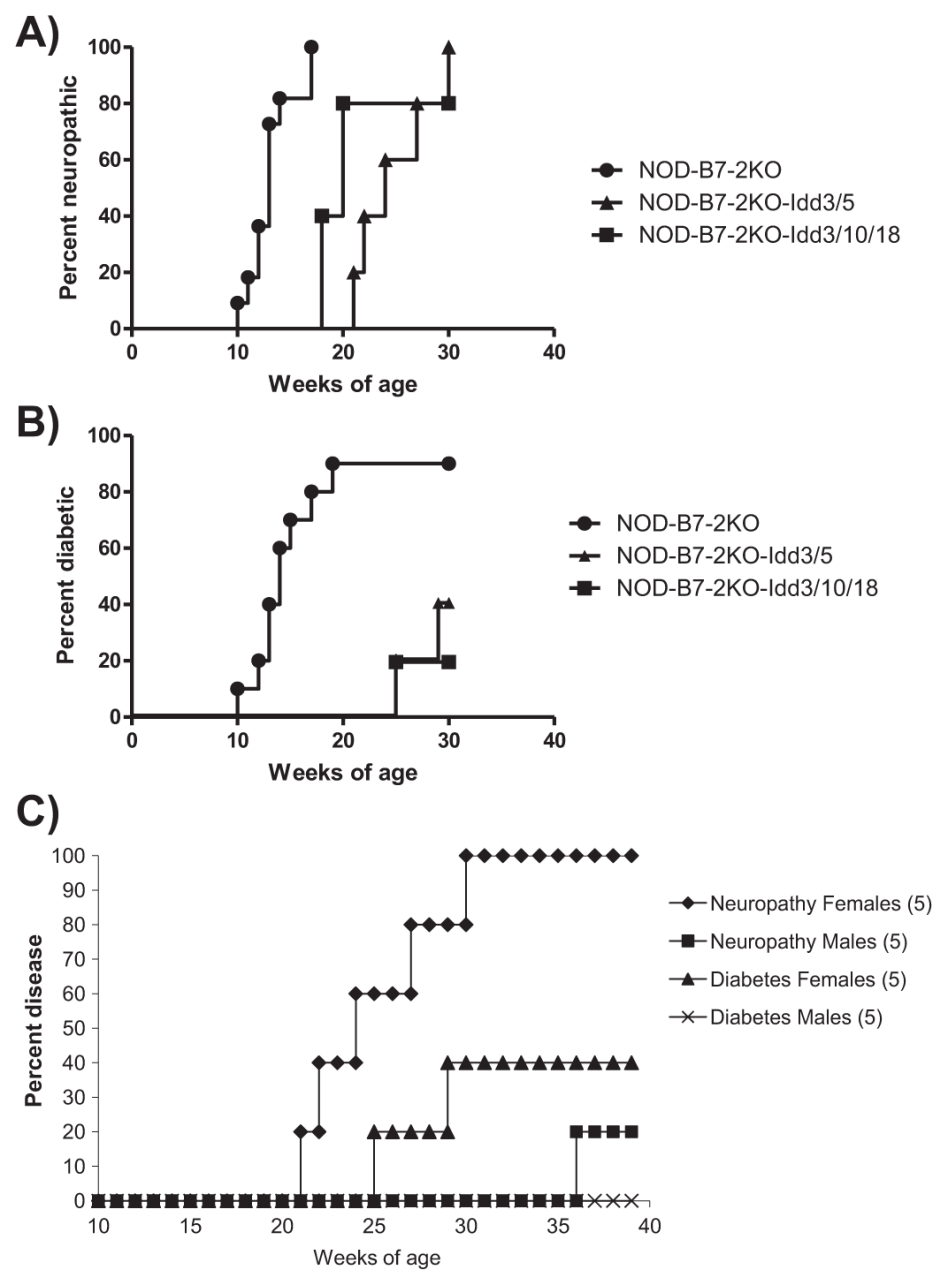
Treatment with anti-B7-1 mAbs restores neuropathy but not diabetes in NOD-B7-2KO-*Idd3*/*5* and NOD-B7-2KO-*Idd3*/*10*/*18* congenic mice. NOD-B7-2KO-*Idd3*/*5* and NOD-B7-2KO-*Idd3*/*10*/*18* congenic mice as well as NOD-B7-2KO controls were treated with anti-B7-1 mAbs between 2–4 weeks of age. A–B) Cumulative incidence of neuropathy (A) and diabetes (B) in female NOD-B7-2KO-*Idd3*/*5* (*n* = 5), NOD-B7-2KO-*Idd3*/*10*/*18* (*n* = 5), and NOD-B7-2KO (*n* = 12) mice is shown. C) Cumulative incidence of neuropathy and diabetes in anti-B7-1 mAbs-treated NOD-B7-2KO-*Idd3*/*5* males and females.

We previously showed that treatment of NOD-B7-2KO mice with anti-B7-1 mAbs between 2 and 4 wk of age accelerated neuropathy and restored diabetes, reflecting a breakdown in immune regulation likely due to reduced numbers of regulatory T cells (Tregs) [29,46]. Since anti-B7-1-treated NOD-B7-2KO mice develop diabetes and neuropathy with similar incidence and kinetics, we took advantage of this model to compare the influence of *Idd3*/*5* and *Idd3*/*10*/*18* loci combinations on each disease within the same animal. As expected, 80–100% of anti-B7-1-treated NOD-B7-2KO mice became both diabetic and neuropathic between 10 and 20 weeks of age (Fig. 4A and B). Furthermore, whereas NOD-B7-2KO-*Idd3*/*5* and NOD-B7-2KO-*Idd3*/*10*/*18* were protected from neuropathy and diabetes (Fig. 3A and data not shown), anti-B7-1 treatment restored neuropathy in both congenic strains but had only a minimal effect on diabetes (Fig. 4A and B). Indeed, 5 out of 5 anti-B7-1-treated NOD-B7-2KO-*Idd3*/*5* and 4 out of 5 NOD-B7-2KO-*Idd3*/*10*/*18* females became neuropathic by 30 weeks of age, but only 2 out of 5 and 1 out of 5, respectively, developed diabetes by 30 weeks of age. Thus, *Idd3*/*5* and *Idd3*/*10*/*18* loci can differentially influence autoimmunity in a tissue-specific manner on the NOD background.

We previously reported that neuropathy and diabetes occurred with similar kinetics and incidence in NOD-B7-2KO males and females treated with anti-B7-1 mAbs [29,46]. In contrast, we observed a strong gender bias in the development of autoimmunity in NOD-B7-2KO-*Idd3*/*5* treated with anti-B7-1 mAbs (Fig. 4C). Whereas 5 out of 5 anti-B7-1-treated NOD-B7-2KO-*Idd3*/*5* females became neuropathic and 2 out of 5 developed diabetes, diabetes and neuropathy were clinically detectable in 0 out of 5 males by 35 weeks of age. This result may reflect a different tropism of auto-immunity in anti-B7-1-treated NOD-B7-2KO-*Idd3*/*5* males vs. females. Indeed, clinical disease was associated with infiltration in peripheral nerves and pancreatic islets as well as severe infiltration in salivary glands in females.

### 3.5. Anti-B7-1 treatment differentially affects the development of autoimmune neuropathy versus thyroiditis in NOD-B7-2KO and NOD-B7-2KO-H-2^h4^ mice

**Fig. 5:**
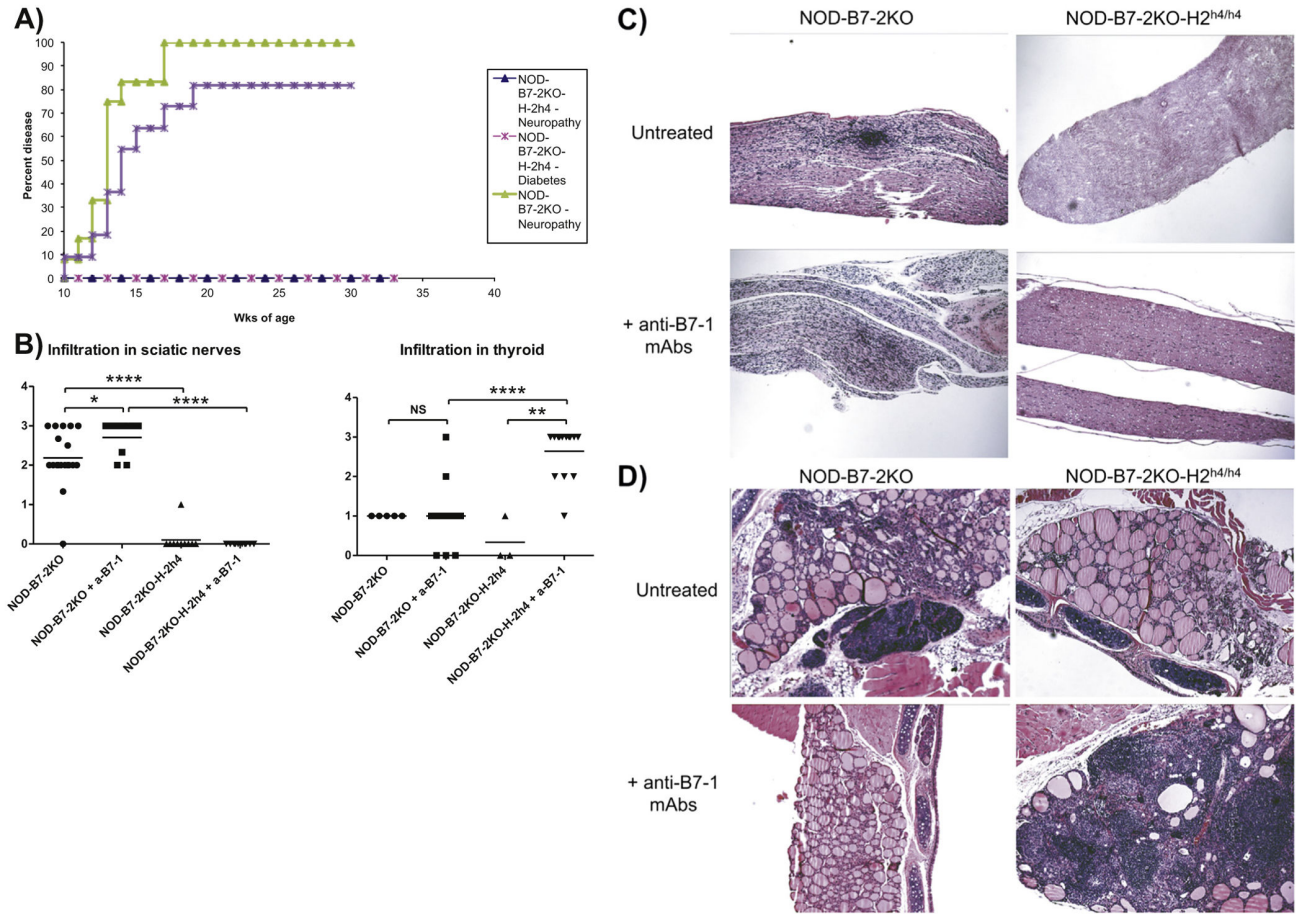
Treatment with anti-B7-1 mAbs does not alter neuropathy and diabetes but exacerbates autoimmune thyroiditis in NOD-B7-2KO-H-2^h4^ congenic mice. NOD-B7-2KO-H-2^h4^ and NOD-B7-2KO controls were treated with anti-B7-1 mAbs between 2–4 weeks of age. A) Cumulative incidence of neuropathy and diabetes in NOD-B7-2KO-H-2^h4^ and NOD-B7-2KO mice. Similar results were observed in males and females and were pooled. B) Sciatic nerves and thyroid glands were isolated from indicated mice, stained with H&E and scored for infiltration as indicated in Materials and methods. Statistical comparison of infiltration severity was performed using the Mann–Whitney test (*: *p* < 0.05; **: *p* < 0.005, ****: *p* < 0.0001). C–D) Representative sections of sciatic nerves (C) and thyroid glands (D) are shown for indicated mice.

In view of data in the previous section suggesting that Treg depletion could supersede the previously established genetic control of a given disease in the NOD background, we set out to examine whether this would still apply in the case of the MHC locus given its disproportionate contribution to susceptibility to autoimmunity. Therefore, we treated NOD-B7-2KO-H-2^h4^congenic mice and NOD-B7-2KO controls with anti-B7-1 mAbs between 2 and 4 weeks of age and followed mice for the development of autoimmunity. We chose to perform these experiments in the NOD-B7-2KO-H-2^h4^strain since, besides being protected from diabetes, NOD-H-2^h4^ mice spontaneously develop autoimmune thyroiditis and represent a model of Hashimoto’s thyroiditis in humans [47,48]. We found that neither neuropathy nor diabetes developed by 30 weeks of age in NOD-B7-2KO-H-2^h4^ mice after anti-B7-1 treatment, in contrast to NOD-B7-2KO mice which developed both diseases with 80–100% penetrance by 15-20 weeks of age (Fig. 5A). Moreover, histological analyses indicated that NOD-B7-2KO-H-2^h4^ mice were protected from mononuclear infiltration in sciatic nerves (Fig. 5B and C) and pancreatic islets (data not shown), and this protection was unaffected by treatment with anti-B7-1 mAbs (Fig. 5B and C). However, anti-B7-1 treatment did have strong effects on autoimmunity in NOD-B7-2KO-H-2^h4^ mice, as revealed by H&E staining of the thyroid gland showing that autoimmune thyroiditis was dramatically exacerbated in anti-B7-1-treated NOD-B7-2KO-H-2^h4^ mice-compared to untreated NOD-B7-2KO-H-2^h4^controls (Fig. 5B and C). Thyroid lesions were also significantly more severe in anti-B7-1-treated NOD-B7-2KO-H-2^h4^ mice compared to anti-B7-1-treated NOD-B7-2KO mice, in agreement with the strong contribution of H-2^h4^ elements to the development of autoimmune thyroiditis on the NOD background. As expected, NOD-B7-2KO mice demonstrated severe infiltration in peripheral nerves but little evidence of thyroiditis (Fig. 5B and C). Treatment with anti-B7-1 mAbs further exacerbated neuritis and restored insulitis (not shown) and diabetes in NOD-B7-2KO mice but failed to induce significant inflammation in the thyroid. Taken together, these results suggest that defective immunoregulation can severely worsen underlying autoimmune responses but does not alter the tissue tropism dictated by genetic elements in the MHC region.

## 4. Discussion

In this study, we generated NOD-B7-2KO strains congenic at various *Idd* loci in order to assess whether autoimmune diabetes and neuropathy were controlled by common or distinct susceptibility genes in the NOD background. We conclude that there is a partial overlap in the genetic control of diabetes and neuropathy that may reveal common defects and pathways leading to auto-immunity. However, we also observed disease-specific features that highlight complex interactions between susceptibility genes, immunoregulation, and tissues targeted by autoimmunity. The NOD H-2^g7^ MHC region appeared necessary for development of both diabetes in NOD mice and neuropathy in NOD-B7-2KO mice. In contrast, other *Idd* loci that strongly influence diabetes did not affect neuropathy when considered individually, and even *Idd* combinations that completely protect NOD mice from disease had a less profound effect on neuropathy. Finally, we uncovered that resistance to disease provided by non-autoimmune gene segments at distinct *Idd* loci was differentially overcome by defective immunoregulation in a tissue/disease-specific manner. Thus, our study helps identify *Idd* loci that control tissue-specific disease or confer general susceptibility to autoimmunity, and brings insight to the Treg-dependence of autoimmune processes influenced by given *Idd* region.

Infiltration into sciatic nerves and development of peripheral neuropathy were completely prevented in NOD-B7-2KO mice when NOD H-2^g7^MH Calleles were replaced by H-2^h4^ or H-2^b^ alleles. The dependence of autoimmune neuropathy on the H-2^g7^ MHC region was not surprising since it is also the most important susceptibility region for autoimmune diabetes in mice and in humans. In particular, *Idd1*, which encompasses the NOD H-2^g7^ region, is the only single locus that is necessary on its own for development of autoimmune diabetes in NOD mice [18]. The mechanisms by which MHC molecules control autoimmunity are still not understood but recent advances in the molecular characterization of peptide-MHC complexes targeted by autoreactive CD4+ T cells in type 1 diabetes (T1D) and other autoimmune diseases have shed some light on this issue [49]. In NOD mice, crystal structure studies demonstrated that the binding-groove of I-A^g7^ molecules was permissive for binding of unique peptide motifs in comparison to other MHC class II molecules that do not predispose to autoimmunity [50,51]. This is significant because the fine features of peptide binding and molecular pathway of peptide presentation by MHC molecules are now believed to affect the selection of autoreactive T cells in the thymus and their activation in the periphery [52,53]. Thus, the dependence of autoimmune neuropathy on the H-2^g7^ MHC in NOD-B7-2KO mice could reflect altered presentation of self-peptides that compromises thymic negative selection of T cells specific for peripheral nerve antigens. In this regard, myelin P0 is an important auto-antigen in spontaneous and induced peripheral neuropathy [30], and development of autoimmune neuropathy in NOD mice carrying a mutant allele of Aire was associated with reduced expression of P0 in medullary thymic epithelial cells (mTECs) and loss of tolerance to P0 [34]. The strong association of the H-2^g7^ MHC with both autoimmune diabetes and neuropathy indicate that these genes may be associated with a general propensity to autoimmunity. It is reminiscent of the association of given HLA alleles with multiple autoimmune diseases in humans, such as the association of the DR3-DQB1*0201 haplotype with T1D, Addison’s disease, Graves’ disease, and Hashimoto’s thyroiditis [54]. However, one notable difference between T1D in NOD mice and autoimmune neuropathy in NOD-B7-KO mice is that heterozygous mice for the MHC region are completely protected from diabetes but not neuropathy, although neuropathy is significantly delayed in heterozygous mice. This suggests that either non-autoimmune MHC alleles are protective for T1D but not neuropathy, or H-2^g7^ MHC-linked susceptibility genes are dominant for both disease but less penetrant for diabetes. Finally, it is worth noting that NOD-H-2^b^ mice deficient for the immunoregulatory molecule PD-1 were previously reported to develop autoimmune peripheral neuropathy and other autoimmune diseases [32,55,56], demonstrating that the H-2^b^ MHC allows the selection of neuropathic T cells. Thus, the fact that NOD-B7-2KO-H-2^b/b^ mice do not develop neuropathy indicates that neuropathy in NOD-B7-2KO mice does not develop as a result of B7-2 deficiency being selectively permissive for neuropathic T cells being selected by any given MHC haplotype.

The occurrence of neuropathy in NOD-H-2^b^-PD-1KO but not PD-1KO mice on a B6 or BALB/c background suggests that susceptibility genes in non-MHC loci control the development of disease, at least when it is mediated by H-2^b^-restricted neuropathic T cells. We examined if autoimmune neuropathy is influenced by a number of *Idd* loci that individually have a strong impact on T1D in NOD mice, in that breeding resistance alleles at any of these single loci reduces incidence of diabetes by 30 to 40 up to 100% with various effects on insulitis. We found that neuropathy developed with normal kinetics and incidence in NOD-B7-2KO mice congenic for *Idd3*, *Idd4*, *Idd5*, *Idd9* or *Idd10*/*18*. Of note, we did not examine all *Idd* loci and it is possible that susceptibility genes in *Idd* loci not tested here could have a comparable influence on diabetes and neuropathy. Nevertheless, it is clear from our data that differences exist in the influence on non-MHC genes on neuropathy versus diabetes in the NOD background. Our results suggest that either the genetic control of diabetes and neuropathy by non-MHC genes is completely distinct or individual susceptibility genes at these loci have different levels of penetrance for each disease. A distinct genetic control of diabetes and neuropathy would be consistent with our previous report that the effector mechanisms involved in each disease were different [29], although susceptibility genes often influence the processes leading to autoimmunity rather than mechanisms involved in tissue destruction *per se*. Moreover, this is in partial agreement with a study by Jiang et al who defined non-MHC quantitative trait loci (QTLs) that modify autoimmune phenotypes other than T1D occurring in NOD-H-2^b^-PD-1KO mice, including peripheral neuropathy [55]. They identified seven QTLs for peripheral neuropathy and neuritis but only three out of seven overlapped with known *Idd* loci, namely *Idd2*, *Idd9* and *Idd15*, indeed suggesting a different genetic basis for neuropathy versus diabetes. In contrast with this report, we did not observe an effect of *Idd9* on neuropathy. This discrepancy could be due to the size of genetic intervals since the described QTL overlaps with *Idd9* but may include additional genes. Moreover, although the immunopathology of neuropathy in NOD-B7-2KO versus NOD-H-2^b^-PD-1KO looked similar overall, it is possible that disease is controlled by a different set of genes in each strain, especially since the strains harbor different MHC alleles and deficiencies in distinct costimulatory pathways, which all could greatly impact the thymic selection of autoreactive T cells and their activation in the periphery.

Despite the fact that individual *Idd* loci tested had no effect on neuropathy in NOD-B7-2KO mice, we observed that both neuritis and clinical disease were almost completely abrogated in NOD-B7-2KO-*Idd3*/*10*/*18*, and NOD-B7-2KO-*Idd3*/*5* mice developed severe neuritis but not neuropathy. Thus, protective alleles for T1D on chromosomes 1 and 3 also control autoimmune neuropathy but require interactions between multiple loci to have an effect on neuritis and clinical disease. This is reminiscent of the control of insulitis in NOD mice. Indeed, while insulitis was linked only to chromosomes 1 (*Idd5*) and 3 (*Idd3* and *Idd10*/*18*) in the original genome scan for susceptibility genes for diabetes [57,58], none of these loci can independently protect from insulitis and interactions between these loci are necessary to reduce insulitis [23,25,26]. In NOD-B7-2KO mice, interactions between the *Idd3* and *Idd10*/*18* protective alleles appeared particularly potent since they could almost completely abrogate both inflammation in peripheral nerves and development of neuropathy. The fact that protective alleles at both *Idd3* and *Idd5* did not reduce neuritis but prevented disease as efficiently as *Idd3*/*10*/*18* combination suggest that there are discrete checkpoints in the development of autoimmune neuropathy, similar to T1D [59–61], and that susceptibility genes in distinct loci can differentially control individual checkpoints. Moreover, our data indicate that a common locus can sometimes interact with multiple other loci with a variable outcome on different checkpoints. Finally, the similar protection for diabetes and neuropathy provided by *Idd3*/*5* and *Idd3*/*10*/*18* loci indicates there is a partial overlap in the genetic basis of these two autoimmune diseases on the NOD background. This is reminiscent of the results of several genome wide associations studies (GWAS) that recently described a number of susceptibility genes associated with a variety of autoimmune diseases, such IL-2, the IL-2 receptor alpha chain (CD25), CTLA-4, and PTPN2, a phosphatase involved in downstream signaling of the IL-2 receptor [62–66].

Candidate genes have been described for *Idd* loci on chromosomes 1 and 3. There is strong evidence that the *Il2* gene is *Idd3*. NOD susceptible alleles at *Idd3* result in reduced IL-2 production compared to B6 protective alleles [24], which has been shown to negatively impact immunosuppression by Tregs and promote the development of diabetes [67,68]. Peripheral neuropathy is controlled by Tregs in NOD-B7-2KO mice since Treg depletion following treatment with anti-B7-1 mAbs results in exacerbated disease. In addition, effects of *Idd3* may be compounded in NOD-B7-2KO mice since interaction of B7-2 with CD28 has consequences on both activation of autoreactive effector T cells (and thus IL-2 production) and homeostasis of Tregs [46]. *Idd3* is nevertheless insufficient by itself to control neuropathy, but interacts with *Idd5* and *Idd10*/*18* regions to provide protection. Among the two candidate genes at *Idd5.1*, i.e. *Ctla4* and *Icos*, accumulating data suggest that *Ctla4* is responsible for the effect of *Idd5.1* on diabetes [39,69]. The NOD susceptibility allele at *Idd5.1* correlates with low levels of the ligand-independent form of CTLA-4 (liCTLA-4), which can suppress T cell responses despite its lack of a B7-binding domain [62,70,71]. Introduction of the liCTLA-4 isoform in NOD mice reduced the incidence and severity of disease [69,72]. Although we cannot exclude the role of other genes, notably at *Idd5.2* and *Idd5.3*, protection from disease but not neuritis suggest that interactions between immune pathways involving IL-2 and CTLA-4 are important to prevent infiltration in peripheral nerves to evolve into tissue destruction and clinical disease. Since CTLA-4 is important both to inhibit conventional T cells and promote Treg function [73], it is possible that these interactions occur within one cell subset or between different cell subsets. Finally, a likely candidate for Idd10 is *Cd101* [74]. CD101 is expressed by multiple cell types, including Tregs, effector T cells and dendritic cells. In particular, B6 protective alleles at Idd10 result in increased expression of CD101 in Tregs compared to NOD alleles and CD101 expression levels on Tregs correlate with their suppressive function [74,75]. While additional studies would be needed to confirm that protection from neuritis and neuropathy in NOD-B7-2KO-*Idd3*/*10*/*18* mice results from interactions between *Idd3*/*Il2* and *Idd10*/*Cd101*, it is tempting to speculate that these interactions could improve both the number and function of Tregs and that protection by *Idd3*/*10*/*18* is thus largely Treg-mediated.

To directly compare the impact of protective *Idd3*/*5* and *Idd3*/*10*/*18* alleles on neuropathy versus diabetes, we took advantage of the development of diabetes and neuropathy in NOD-B7-2KO mice treated with anti-B7-1 mAbs between 2 and 4 wk of age [29,46]. We previously showed that the accelerated and concomitant development of both diseases in anti-B7-1-treated NOD-B7-2KO mice was likely due to defective immunoregulation stemming from reduced numbers of Tregs, although a role for abrogated CTLA-4 signaling has not been excluded. Here, we found that treatment of NOD-B7-2KO-*Idd3*/*5* and NOD-B7-2KO-*Idd3*/*10*/*18* congenic mice with anti-B7-1 mAbs restored neuropathy but not diabetes. This indicates that the protection afforded by *Idd3*/*5* and *Idd3*/*10*/*18* is stronger for diabetes than neuropathy, suggesting that susceptibility genes at these loci play a more integral role in autoimmune responses targeting pancreatic islets compared to peripheral nerves. Of note, the fact that NOD-B7-2KO-*Idd3*/*5* and NOD-B7-2KO-*Idd3*/*10*/*18* are still protected from diabetes after treatment with anti-B7-1mAbs, i.e. in a model where diabetes occurs as a consequence of a defective Treg compartment, suggests that *Idd3*/*5* and *Idd3*/*10*/*18* alleles are protective for diabetes in a Treg-independent manner. In contrast, Treg deficiency abrogated the protective effect of *Idd3*/*5* and *Idd3*/*10*/*18* on neuropathy, which is consistent with interaction of candidate genes at these loci having a protective effect on neuropathy due to a major impact on Tregs. Surprisingly, the protective effect of *Idd3*/*5* on sialadenitis was also abrogated after anti-B7-1 treatment. This was unexpected since NOD susceptibility alleles at *Idd3* and *Idd5* were previously shown to be necessary and sufficient for the development of sialadenitis [76,77]. This result suggests that given genes and pathways that control autoimmunity in the NOD mouse may be overridden in conditions of defective immunoregulation in a tissue/disease-specific manner. Conversely, the effects of MHC alleles on disease were unaffected by Treg depletion, as evidenced by the clear tissue polarization in NOD-B7-2KO versus NOD-B7-2KO-H-2^h4^ mice that remains unaffected after anti-B7-1 treatment. Namely, anti-B7-1-treated NOD-B7-2KO mice develop neuropathy and diabetes but not thyroiditis, whereas anti-B7-1-treated NOD-B7-2KO-H-2^h4^ mice develop severe thyroiditis but not diabetes or neuropathy. Of note, thyroiditis did not appear to develop in untreated NOD-B7-2KO-H-2^h4^ mice, even after 60 wks of age. This differs from the NOD-H-2^h4^ strain, in which two-third of animals spontaneously develop thyroid lesions after 6 months of age without iodine supplementation in the drinking water [48], and likely reflects the fact that B7-2 deficiency prevents a number of autoimmune syndromes that normally develop in NOD mice, such as diabetes and sialadenitis [4,78]. The dramatic increase in the severity of thyroiditis in anti-B7-1-treated NOD-B7-2KO-H-2^h4^ mice compared to untreated mice is consistent with the previously reported role of Tregs in controlling thyroiditis in NOD-H-2^h4^ mice [79,80].

In conclusion, we showed that autoimmune neuropathy and diabetes on the NOD background have an overlapping but distinct genetic basis. Additionally, we found potent genetic interactions of some *Idd* loci that provided almost complete protection from neuritis and clinical neuropathy, and further showed that defective immunoregulation by Tregs could supersede protection by some, but not other, *Idd* loci in a tissue-specific manner. Taken together, these findings have implications for the identification of susceptibility genes for autoimmune peripheral neuropathy. Moreover, our study uncovers that the influence of protective alleles on autoimmune diseases can be differentially affected by Treg defects depending on the tissue target by autoimmunity, indicating the complexity of defining the genetic basis of auto-immune diseases.

Finally, this paper is part of a dedicated issue of the Journal of Autoimmunity devoted in this case to Professor Abul Abbas, part of the Journal’s mission within the last several years to highlight distinguished immunologists. In my case, I have known Abul for many years, but have only begun to appreciate his rock star talents during the past dozen years at the University of California at San Francisco. Abul, you have an amazing ability to make things simple for the beginning immunologist while performing in depth research that transforms our understanding of immunology. Your multiple books and lectures around the world have influenced so many and created passion among immunology researchers very few people in all of science can match. The rock star designation is apropos and the only thing I am missing is your autograph on a golf ball.
